# Intra-Bone Marrow Administration of miR-140-3p Improves Bone Metabolism in a Growing Senescence-Accelerated Mouse Prone 6 Strain

**DOI:** 10.3390/biomedicines13040883

**Published:** 2025-04-05

**Authors:** Hironobu Katsuyama, Kazue Tanda, Kumiko Terada, Shigeko Fushimi, Takeshi Yoda, Vitalii Katsuyama, Tsutomu Nohno

**Affiliations:** 1 Department of Public Health, Kawasaki Medical School, Kurashiki 701-0192, Japantyoda@med.kawasaki-m.ac.jp (T.Y.); tnohno@gmail.com (T.N.); 2 Department of Natural Sciences, Kawasaki Medical School, Kurashiki 701-0192, Japan; 3 Department of Medical Welfare, Kawasaki University of Medical Welfare, Kurashiki 701-0193, Japan

**Keywords:** miR-140-3p, senescence-accelerated mice prone 6 strain, lipid nanoparticles, flotillin 2, bone histomorphometry

## Abstract

**Background**: Our previous study demonstrated that miR-140-3p induced osteocalcin expression in osteoblastic MC3T3-E1 cells. In this study, we investigated the direct effects of miR-140-3p on bone turnover in senescence-accelerated mice. **Methods**: In order to evaluate the effects of miR-140-3p, we formulated lipid nanoparticles (LNPs) containing miR-140-3p (100 μg/mL), with or without flotillin-2 (Flo2), a microvesicle marker excreted by osteoblasts. LNP was administered into the right tibia of the P6 strain of senescence-accelerated mice (SAMP6). Four-week-old SAMP6 males were divided into three groups: control, LNP, and LNP + Flo2. LNPs were administered five times, once every three days. No gait abnormalities were observed in any group. Two days after the last administration of LNPs, blood and urine samples were collected to measure bone turnover markers and blood chemistry and to perform urinalysis. Bone histomorphometry was performed on the left femur, contralateral to the administration site. The pancreas was removed for insulin staining of the Langerhans islets. **Results**: The LNP + Flo2 group showed greater bone volume, trabecular thickness, and osteoid thickness in bone histomorphometry. Carboxylated osteocalcin, a bone formation marker, was also higher in the LNP + Flo2 group, indicating that LNP + Flo2 activated osteoblastic function. Insulin levels in the islets of Langerhans did not differ across the groups, consistent with under-carboxylated osteocalcin levels. **Conclusions**: LNP + Flo2 effectively improved bone metabolism.

## 1. Introduction

Osteoporosis is one of the major health problems worldwide as it increases the risk of fractures, which can leave older people bedridden [1]. In order to prevent osteoporotic fractures, it is essential to maximize bone mass during adolescence. Bone turnover is a continuous process in which bone is resorbed by osteoclasts and formed by osteoblasts to maintain bone mass [2]. Bone turnover is tightly regulated by multiple signaling pathways. For instance, osteoblast differentiation is regulated by the WNT/β-catenin signaling [3] pathway, the bone morphogenetic protein (BMP)-Smad pathway [4], and others, while osteoclast differentiation is regulated by the receptor activator of the nuclear factor κB (RANK)-RANK ligand pathway [5], among others. Recent evidence indicates that microRNAs (miRNAs) regulate multiple cellular functions by inhibiting mRNA translation [6], miR-204/211 inhibits *RUNX2* transcription, miR-637 inhibits *osterix* transcription in osteoblasts, and miR-124 inhibits *c-FOS* transcription in osteoclasts [7], among others. Our previous study demonstrated that the Wnt3a signaling pathway activated transforming growth factor β3 (TGFβ3) and inhibited *miR-140-3p* expression in osteoblastic MC3T3-E1 cells, and transfecting miR-140-3p into osteoblastic MC3T3-E1 cells suppressed *TGFβ3* expression while activating *osteocalcin* (*OCN*) expression [8].

Senescence-accelerated mouse (SAM) strains were developed in the early 1970s through repeated inbreeding, with breeding being selected based on shortened lifespan and accelerated aging [9]. SAM-prone strains are classified into 11 groups according to their predisposition to senile diseases. SAMP1, 2, 7, and 11 showed senile amyloidosis; SAMP3 showed temporomandibular disorder; SAMP6 showed osteoporosis; SAMP8 and SAMP10 showed learning and memory impairment; and SAMP9 showed cataracts [10,11,12]. SAMP6 showed decreased bone mineral density (BMD) and short lifespan [13]. Our previous study demonstrated that menaquinone-4, a vitamin K2 subtype, reversed water-immersion restraint stress-induced bone loss of SAMP6 [14].

OCN, produced by osteoblasts, is the most abundant non-collagenous protein in bone tissue [15]. OCN is modified by γ-glutamyl carboxylase (GGCX) into carboxylated OCN called Gla. Gla is decarboxylated while bone resorption is processed, becoming under-carboxylated OCN called Glu. The effects of OCN remain controversial as OCN is essential for maintaining muscle mass in aged mice [16], while undercarboxylated OCN is associated with muscle strength and bone health in elderly women [17]. In contrast, OCN is required for apatite crystallite alignment but not for glucose metabolism, testosterone synthesis, or muscle mass [18].

Microvesicle (MV) is a novel mechanism to communicate with osteoblasts and osteoclasts [19]. Parathyroid hormone enhances not flotillin-2 (Flo2) but *RANKL* expression in MVs. Although Flo2 is a MV marker of osteoblasts, whether Flo2 enhances the function of miRNA is unknown.

In this study, miR-140-3p-loaded lipid nanoparticles (LNPs) were prepared and directly injected into bone marrow, and their effects were investigated in SAMP6. This is the first study to administer miR-140-3p to SAMP6.

## 2. Materials and Methods

### 2.1. Preparation of LNP Containing miR-140-3p

LNP was formulated containing miR-140-3p (100 μg/mL) with or without Flo2, an MV marker secreted by osteoblasts [19]. These LNPs were manufactured by Katayama Chemical Industries Co., Ltd. (Osaka, Japan).

### 2.2. Animals and Experimental Protocol

**Figure 1:**
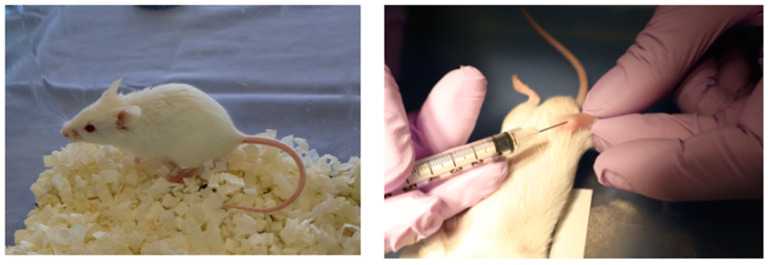
SAMP6 mouse and intra-bone marrow injection. Intra-bone marrow injection was performed in the right tibia.

Three-week-old male SAMP6 mice were obtained from Japan SLC, Inc. (Hamamatsu, Japan). Mice were housed under controlled conditions (22.2 °C, constant humidity, 12 h light/dark cycle) with standard rodent chow and tap water. Animals were acclimated to these conditions for 1 week. Mice were then divided into three groups (*n* = 3 per each group) based on initial body weight: control, LNP, and LNP + Flo2. These entire experiments were performed twice. Intra-bone marrow administration was performed according to Takeda’s method [20]. Briefly, mice were anesthetized, the right knee was flexed to 90 degrees, and the proximal side of the tibia was drawn to the anterior. A 26-gauge needle was inserted into the joint surface of the right tibia through the patellar tendon and the inserted into the bone marrow cavity of the right tibia. Intra-bone marrow administration of saline solution, LNP, or LNP + Flo2 10 μL each was performed five times at three-day intervals (Figure 1).

The body weight of each animal was measured at the time of each injection until the final day of LNP administration. To assess bone formation, mice were double-labeled with two subcutaneous injections: tetracycline hydrochloride (TL) (20 mg/kg) and calcein (CL) (10 mg/kg; Sigma-Aldrich Corp., St. Louis, MO, USA) at 5 and 2 days before euthanasia, respectively. Mice were housed in metabolic cages, and urine samples were collected 16 h after the final administration. Urine samples were stored at −80 °C until analysis. One day after the final LNP administration, mice were anesthetized with sevoflurane, and serum samples were collected using MiniCollect serum tubes (Greiner Bio-One, Kremsmünster, Austria) and stored at −80 °C until analysis. After blood collection, left femurs were removed, cleaned of soft tissues, and fixed in 70% ethanol for bone histomorphometry. The right femurs were removed for BMD measurement. Pancreases were removed for insulin staining. The experimental protocol was approved by the Animal Research Committee of the Kawasaki Medical School (#24-033) in accordance with the ARRIVE guidelines.

### 2.3. Biochemical Markers and Bone Turnover Markers

Serum alkaline phosphatase (ALP), total protein (TP), calcium (Ca), inorganic phosphorus (IP), glucose, and hemoglobin A1c (HbA1c) levels, as well as urinary creatinine (U-Cre) and urinary calcium (U-Ca), were measured at Nagahama Life Science Laboratory, Oriental Yeast Co. Ltd. (Nagahama, Japan). Serum levels of Gla-osteocalcin and Glu-osteocalcin, a bone formation marker, were determined using mouse Gla-Osteocalcin High Sensitive EIA and mouse Glu-Osteocalcin High Sensitive EIA Kits (MK127 and MK129, TAKARA Biomedicals, Kyoto, Japan). Bone resorption markers, including serum tartrate-resistant acid phosphatase-5b (TRACP 5b) and urinary C-terminal telopeptides of type I collagen (CTX), were assessed using a mouse TRAP^TM^(TRAcP5b) ELISA (SB-TR103, Immunodiagnostic Systems, through Filgen, Inc., Nagoya, Japan) and Rat-Laps@(CTX-I) EIA (AC-06F1, Immunodiagnostic Systems, through Filgen, Inc., Nagoya, Japan). Urinary CTX level was corrected by urinary Cre concentration.

### 2.4. Trabecular (Tb) BMD

The fixed right femoral bones were analyzed using a small-animal X-ray computed tomography system (LaTheta LCT-200; Aloka, Osaka, Japan). Each bone was placed horizontally inside a tube and scanned using a 96 μm voxel. The scan line was adjusted using the scout view. Tb BMD was measured in the secondary spongiosa, 3 mm proximal to the distal femoral growth plate. The data were quantified using the automated image analysis software provided with the device.

### 2.5. Bone Histomorphometry

Bone histomorphometry was performed on the secondary spongiosa of the left femoral distal end of the samples at the Ito Bone Histomorphometry Institute (Niigata, Japan). The distal femoral end was examined in coronal view using Villanueva bone staining. The histomorphometric bone analysis assessed tissue volume (TV, μm^2^), bone volume (BV, μm^2^), bone surface (BS, μm), double-labeled surface (dLS, mm), Tb thickness (Tb.Th, μm), and the number of osteoclasts (N.Oc, cells) and osteoblasts (N.Ob, cells). Obs were further classified as type II–IV according to morphological criteria [21]: type II, classic cuboidal or columnar with adjacent nuclear clear zone; type III, intermediate without adjacent nuclear clear zone; and type IV, lining transitional cytoplasm that is extremely thin and a undulating line (most mature population) [22]. However, type I Obs cannot be detected by microscopy. The following parameters were derived from the aforementioned primary parameters: bone volume (BV/TV, %), Tb thickness (Tb.Th, μm), osteoid surface to bone surface ratio (OS/BS, %), eroded surface (ES/BS, %), number of Ocs (N.Oc/BS, N/mm), surface area of Ocs (Oc.S/BS, %), mineral apposition rate (MAR, μm/D), surface area of Ob (Ob.S/BS, %), and number of Obs (N.Ob/BS, N/mm). Standard bone histomorphometric nomenclature, symbols, and units were used as described in the report by the American Society for Bone and Mineral Research Histomorphometry Nomenclature Committee [23].

### 2.6. Immunofluorescence Staining of Insulin in Pancreatic Islets of Langerhans

Removed pancreases were fixed in 4% paraformaldehyde for 24 h and then washed three times with phosphate-buffered saline prior to analysis. Tissue samples were embedded in paraffin, sectioned at 4 μm thickness, and mounted on salinized slides. Sections were incubated with the primary antibody, a rabbit monoclonal anti-insulin IgG 1:10,000 (Abcam, #ab181547, Cambridge, UK). Then they were incubated with the secondary antibody, i.e., the goat anti-Rabbit IgG (H+L) Cross-Absorbed Secondary Antibody (Invitrogen #A-11012, Waltham, MA, USA). Pancreatic islets were visualized at 20× magnification using the All-in-One Fluorescence Microscope (Keyence, Osaka, Japan).

### 2.7. Statistical Analysis

All data were expressed as mean ± standard deviation (SD). One-way analysis of variance (ANOVA), followed by a Turkey–Kramer post hoc test, was performed using EZR software (version 4.2.2) to compare differences among control, LNP, and LNP + Flo2 [24].

## 3. Results

### 3.1. Growth of SAMP6 and Influence of Intra-Bone Injection

We used SAMP6 since osteopenia would occur in at the early stage. No significant differences were observed in initial or final body weights of SAMP6 (Table 1). Furthermore, no gait disturbances were observed in any group following intra-bone marrow injection. Furthermore, femoral lengths did not differ across the groups.

### 3.2. Biochemical Markers of SAMP6

No statistical differences were observed in ALP, insulin, glucose, HbA1c, and U-Ca/U-Cre between control, LNP, and LNP + Flo2 (Table 1). Serum Ca was significantly lower, and TP tended to be lower in LNP + Flo2 than in control and LNP; while IP tended to be higher in LNP than in control and LNP + Flo2. The serum Ca level of LNP + Flo2 was significantly lower than that of the other groups, which might reflect the low level of TP.

### 3.3. Bone Turnover Biochemical Markers and BMD of SAMP6

In bone formation markers, while Glu, the undercarboxylated form of OCN, showed no significant differences across the groups, Gla levels in LNP and LNP + Flo2 were significantly higher than in the control (Table 2). In addition, another bone formation marker, ALP, was not different across the groups (Table 1). In bone resorption markers, TRACP 5b and urinary CTX did not differ across the groups. These findings suggest that LNPs affect bone formation. On the contrary, both the trabecular BMD and the cortical BMD did not differ across the groups.

### 3.4. Primary Measurements of Trabecular Bone of SAMP6

**Figure 2:**
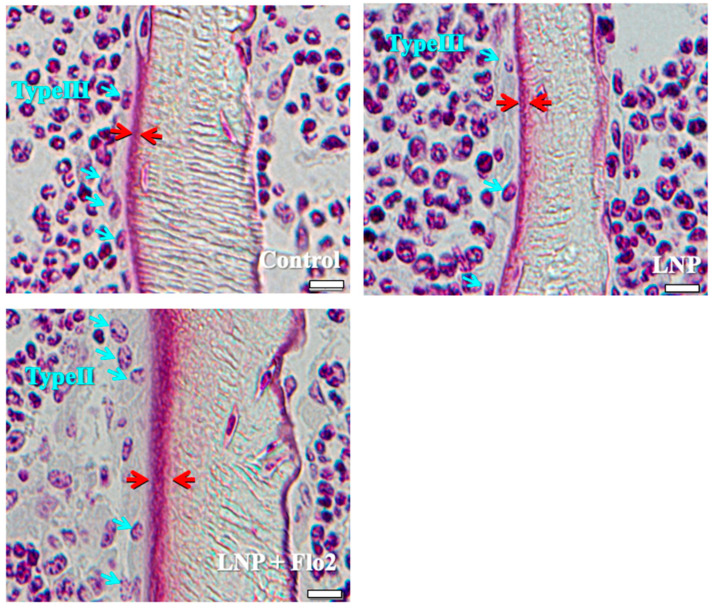
Types of osteoblasts. Control and LNP show type III osteoblasts, and LNP + Flo2 show type II osteoblasts (blue arrow). The red arrow indicates the osteoid thickness. Scale bar: 10 μm.

The number of type II osteoblasts was significantly higher in LNP + Flo2 than in control and LNP, although no statistical differences were observed in type III and type IV osteoblasts (Table 3). Furthermore, trabecular and osteoid thicknesses were significantly higher in LNP + Flo2 than in control and LNP (Table 3). Figure 2 shows that control and LNP showed type III osteoblasts, while LNP + Flo2 showed type II osteoblasts. Since type II osteoblasts are associated with osteoid formation, and type III and type IV osteoblasts are associated with mineralization [2], LNP + Flo2 may lead to osteoid formation.

**Figure 3:**
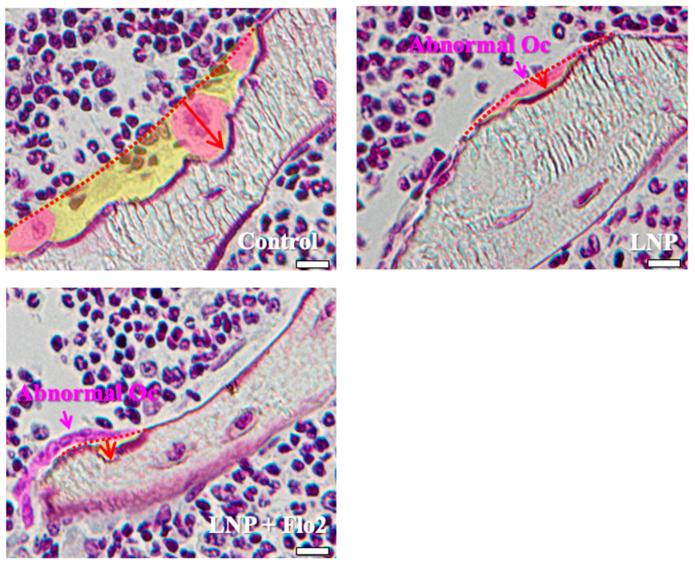
Characteristics of osteoclasts. Control shows one multinucleated osteoclast and two mononucleated osteoclasts, and all of them have rounded nuclei. The resorption area is wide, and the erosion depth is deep. LNP and LNP + Flo2 show abnormal osteoclasts with a flat nucleus and shallow erosion depth. Red arrow: erosion depth; yellow area: resorption area. Scale bar: 10 μm.

Since the numbers of both multinucleated and mononucleated osteoclasts were significantly lower in LNP + Flo2 than in control and LNP, and the number of abnormal osteoclasts was significantly higher in LNPs than in control, the erosion depth was significantly reduced in LNPs compared to control (Table 3, Figure 3). These findings reveal that resorption areas in LNPs were narrower than in control.

### 3.5. Mineralizing Parameters of Trabecular Bone of SAMP6

**Figure 4:**
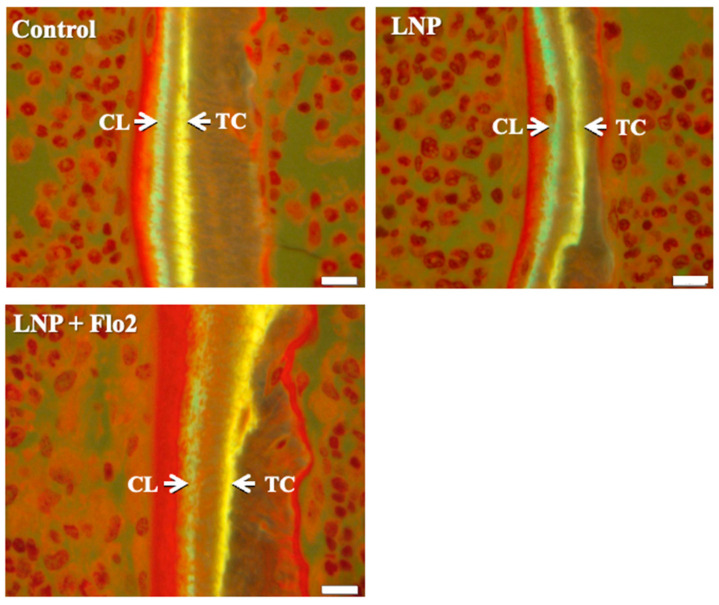
Measurement of mineral apposition. Double staining was performed using TC (yellow) and CL (green). Interval between TC and CL injections is 2 days, and the distances between TC and CL labels are used to calculate the mineral apposition rate (MAR). Scale bar: 10 μm.

BV/TV was significantly higher in LNP + Flo2 than in control and LNP (Table 4). On the contrary, ES/BS was significantly lower in LNP + Flo2 than in control and LNP. Since tetracycline and calcein were used for double labeling, the time interval between the injections of the two labels enabled MAR determination, with wider distances between labeled layers indicating rapid mineralization. LNP + Flo2 showed a significantly higher MAR than control and LNP (Table 4, Figure 4). And BFR/BS was also higher in LNP + Flo2. Although MS/BS did not differ across the groups, Ob.S/BS was significantly higher in LNP + Flo2, and Oc.S/BS was significantly lower in LNP + Flo2. These findings demonstrated that LNP + Flo2 promoted osteoblastogenesis while reducing osteoclastic activity.

### 3.6. Microscopic Observation of Secondary Spongiosa of SAMP6

**Figure 5:**
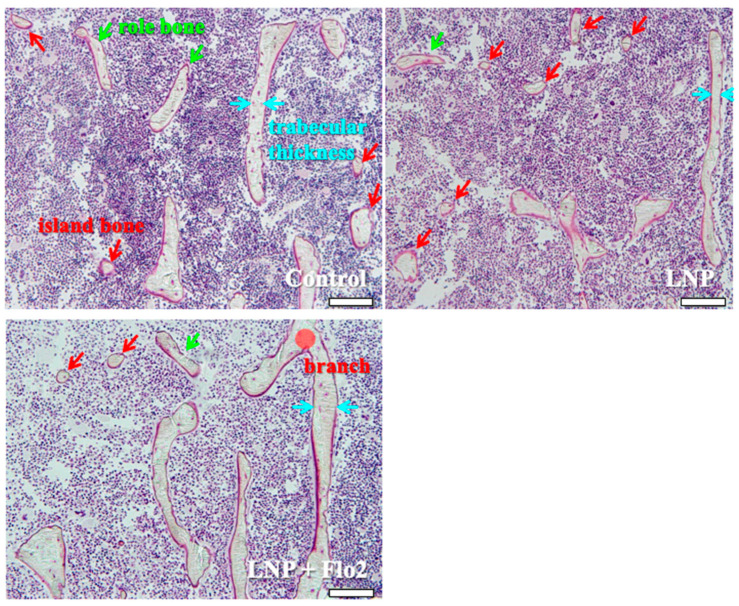
Observation of secondary spongiosa. Control shows island and role bones, which are characteristic of osteopenia. LNP shows many island bones. LNP + Flo2 shows long trabeculae and a trabecular branch. Trabecular thickness is wider than control. Green arrow shows role bone; red arrow shows island bone; and blue arrow shows trabecular thickness. Scale bar: 100 μm.

Secondary spongiosa of control (Figure 5) showed many island and role bones and a thin trabeculae. This phenomenon was typically observed during bone volume reduction and was considered a characteristic of SAMP6. Although LNP also showed the same tendency, LNP + Flo2 showed wide trabeculae and branch.

### 3.7. Immunofluorescence Staining for Insulin in Pancreatic Islets of Langerhans

**Figure 6:**
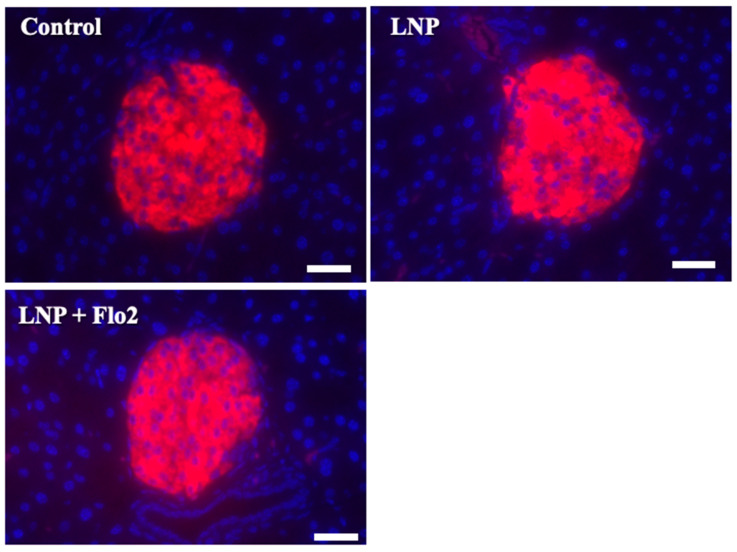
Immunofluorescence staining for insulin in pancreatic islets of Langerhans. Red staining indicates insulin. The insulin signal was calculated by light intensity per islet area. Scale bar: 50 μm.

Since serum insulin levels did not differ across the groups. Immunofluorescence staining for insulin was performed on pancreatic islets of Langerhans (Figure 6). Red staining indicated the insulin signal, which was 73.0 ± 6.8, 73.0 ± 6.7, and 80.8 ± 6.5 per mm^2^ for the control, LNP, and LNP + Flo2 groups, respectively (*n* = 54 for control, *n* = 74 for LNP, and *n* = 69 for LNP + Flo2). Red signals did not differ across the groups.

## 4. Discussion

SAM was developed in the early 1970s as an accelerated senescence model. The original study shows that SAMP6 begins to lose bone at 16 weeks and exhibits significant bone loss by 28 weeks [25]. Since ovariectomized SAMP6 females did not develop osteopenia [13], we used 5-week-old male SAMP6 mice that showed characteristic bone formation in bone histomorphometry and were considered a suitable model for osteopenia. Furthermore, unlike female SAMP6 during adolescence, male SAMP6 was considered hormonally stable. Since intra-bone marrow administration did not result in gait disturbance, this model proved suitable for evaluating osteoblastic function.

MicroRNAs serve as key post-transcriptional regulators of gene expression, playing crucial roles in various cellular functions [26]. Regarding bone remodeling, miR-30d-5p and miR-133b-3p target *RUNX2* and inhibit osteoblastic differentiation in mineralizing MC3T3-E1 cells [27]. Although miR-140-3p targets *BMP2* and inhibits osteoblast differentiation in MC3T3-E1cells [27], miR-140-3p mimic promotes fracture healing through Wnt and β-catenin overexpression in rats [28]. Based on our previous study, in which miR-140-3p induced *OCN* expression by suppressing TGFβ3 in osteoblastic MC3T3-E1 cells [8], we investigated the effects of miR-140-3p on bone and glucose metabolisms in SAMP6. In LNP groups, Gla was significantly higher than control, reflecting the upregulation of osteoblastic function. On the contrary, Glu was not different across the groups, reflecting that osteoclastic functions were downregulated in LNP groups. Although LNP + Flo2 administration increased serum Gla but not Glu, both trabecular and cortical BMD did not differ across the groups. This was because the SAMP6 appeared to be young. Since we measured total ALP, the level of ALP did not seem to be different across the groups. Further study seems to be necessary in order to measure bone-specific ALP. In bone histomorphometry, a number of type II osteoblasts—Tb.Th, O.Th, BV/TV, MAR, and Ob.S/BS—were increased in the LNP + Flo2 group. Differences between bone histomorphometry and BMD indicated that BMD measurement was not sensitive enough to detect changes in bone microarchitecture. Osteoblasts are classified into four groups, i.e., from type I to type IV. Type II osteoblasts are cuboidal and actively produce osteoid; they are so-called active osteoblasts. Type III cells are semi-cuboidal and also active osteoblasts, but they are usually smaller than type II osteoblasts. Type IV cells are called lining osteoblasts; they have a flat nucleus and extremely thin cytoplasm. Type III and IV osteoblasts are associated with mineralization. These osteoblasts change greatly with differentiation and maturation, along with the change in osteoblastic function [21]. These findings suggested that intra-bone marrow administration of LNP + Flo2 improved bone formation, as evidenced by bone histomorphometry, which may lead to increased BMD. Furthermore, the LNP + Flo2 group showed reduced osteoclast numbers and superficial erosion depth, suggesting inhibition of bone resorption. Moreover, since abnormal osteoclasts were observed in LNPs, miR-140-3p might directly influence osteoclast function. Since LNPs were administered to the right tibia, and bone histomorphometry was performed on the left tibia, miR-140-3p may improve bone remodeling through osteoblastic function. Since flotillin 2 (Flo2) is a microvesicle marker of osteoblast-derived exosome [19,27], adding Flo2 to LNP is effective.

OCN, consisting of 46 amino-acids in a mouse model [29], is primarily produced by osteoblasts. Post-translational modification of OCN is performed at glutamate residues in positions of 13, 17, and 20 by γ-glutamyl carboxylase, with vitamin K as a co-factor. Gla is incorporated into the bone matrix, and during bone resorption, Gla is decarboxylated and becomes Glu. Secreted Glu is understood to act as a hormone, influencing processes such as insulin production [29], adiponectin production [30], and testosterone production [29], among others. On the contrary, OCN knockout mice do not show this effect, suggesting that OCN may not have hormonal effects [18]. Our previous study showed that menaquinone-4 (MK-4, a vitamin K2 subtype) improved bone turnover through water-immersion restraint stress-induced bone loss in SAMP6 [14] via osteoblast formation, although no statistical differences were observed in Gla levels. In the present study, Gla levels were higher in LNPs than in control, reflecting improved osteoblastic activity. On the other hand, Glu levels did not differ across the groups. When osteoclasts initiate bone resorption, they secrete TRACP-5b [31], while CTX is released through collagen degradation. In the present study, TRACP-5b and CTX levels did not differ across the groups, while bone histomorphometry showed flattened osteoclasts and narrow bone resorption areas in LNPs. These findings demonstrated that LNPs inhibited osteoclast function.

Patients with diabetes mellitus (DM) have increased bone fragility and risk of osteoporotic fracture [32]. DM-induced bone fragility is associated with increased collagen cross-links of advanced glycation end-products and dysfunction of osteoblasts and osteocytes. In vitro simulations demonstrate that osteoblasts and osteoclasts are negatively impacted in the DM-simulated microenvironment [33]. On the contrary, insulin signaling promotes bone formation through suppression of the osteoblast development inhibitor Twist 2 and enhances osteocalcin expression [34]. Bone and glucose metabolism are thought to be closely related. In the present study, insulin levels remained unchanged in both serum and pancreatic islets of Langerhans, despite the upregulation of the osteoblast function by LNP + Flo2. Further research is needed to elucidate the relationship between miR-140-3p and insulin secretion.

In the present study, miR-140-3p plays a crucial role in bone formation through osteoblastic function. This study had several limitations. First, SAMP6 were too young, making it difficult to accurately assess bone histomorphometry. Older SAMP6 mice, such as those at 16 weeks of age, may show more pronounced differences. Second, although bone histomorphometry was performed on the contralateral side to the injection site, osteoblast activation may have resulted from direct Gla effects, circulating miR-140-3p, or other factors. Third, this study assessed bone metabolism over a relatively short period; further research is warranted to elucidate the long-term effects of miR-140-3p. Fourth, a relatively small sample size (*n* = 6 for each group) was used in the present study; experiments with larger sample sizes should be performed.

## 5. Conclusions

LNP + Flo2, containing miR-140-3p, improves bone formation through osteoblast activation and osteoclast inhibition.

## 6. Patents

We were granted a Japanese patent entitled “Promotion of osteocalcin production by miR-140-3p transfected osteoblasts” (6948059).

## Figures and Tables

**Table 1:** Initial and final body weights and biochemical markers of SAMP6 mice.

Measurement	Control (*n* = 6)	LNP (*n* = 6)	LNP + Flo2 (*n* = 6)
Initial body weight (g)	22.6 ± 2.0	23.4 ± 1.4	23.9 ± 1.5
Final body weight (g)	28.4 ± 2.1	30.2 ± 1.5	30.0 ± 1.1
ALP (U/L)	209.3 ± 13.3	218.2 ± 29.2	202.7 ± 16.1
TP (mg/dL)	5.10 ± 0.14	5.23 ± 0.15	4.98 ± 0.22 #
Ca (mg/dL)	10.13 ± 0.10	10.15 ± 0.18	9.83 ± 0.23 *
IP (mg/dL)	7.03 ± 1.10	8.63 ± 1.47 #	7.23 ± 0.48
Insulin (ng/mL)	6.70 ± 4.42	5.76 ± 3.82	3.68 ± 1.18
Glucose (mg/dL)	186.0 ± 18.5	183.2 ± 27.6	193.5 ± 32.7
HbA1c (%)	3.87 ± 0.09	3.82 ± 0.04	3.81 ± 0.08
U-Ca/U-Cre (mg/mg Cre)	0.30 ± 0.13	0.38 ± 0.18	0.24 ± 0.04

Statistical differences were observed *: *p* < 0.05 between control and LNP + Flo2, LNP, and LNP + Flo2, #: *p* < 0.10 between LNP and LNP + Flo2 (TP), between LNP and control, and between LNP and LNP + Flo2 (IP). All experiments were performed in duplicate.

**Table 2:** Biochemical markers of bone turnover and bone mineral density.

Measurement	Control (*n* = 6)	LNP (*n* = 6)	LNP + Flo2 (*n* = 6)
Glu (ng/mL)	1.81 ± 0.41	1.73 ± 0.18	1.57 ± 0.23
Gla (ng/mL)	53.6 ± 7.6	63.2 ± 4.0 *	64.1 ± 6.7 *
TRACP-5b (U/L)	13.5 ± 1.2	14.3 ± 2.6	14.3 ± 1.4
CTX (ng/mL Cre)	5.21 ± 0.10	5.10 ± 0.67	5.38 ± 1.74
Trabecular BMD (mg/cm3)	293.9 ± 11.5	264.5 ± 36.2	295.3 ± 55.5
Cortical BMD (mg/cm3)	633.1 ± 21.4	649.4 ± 42.7	638.1 ± 26.2

*: *p* < 0.05, statistical differences were observed between control and LNP and between control and LNP + Flo2. All experiments were performed in duplicate.

**Table 3:** Primary measurements of trabecular bone.

Measurement	Control	LNP	LNP + Flo2
N.Ob.Type II/BS (N/mm)	5.96 ± 3.25	7.66 ± 0.82	11.72 ± 3.85 *1
N.Ob.Type III/BS (N/mm)	5.62 ± 3.05	4.75 ± 1.35	5.42 ± 0.56
N.Ob.Type IV/BS (N/mm)	1.81 ± 1.18	1.44 ± 0.52	1.33 ± 0.55
Tb.Th (μm)	27.1 ± 2.6	26.2 ± 2.6	31.2 ± 2.8 *2
O.Th (μm)	2.21 ± 0.08	2.22 ± 0.05	3.00 ± 0.15 *3
N.Mu.Oc/BS (N/mm)	3.49 ± 0.57	2.89 ± 2.31	1.33 ± 0.87 *1
N.Mo.Oc/BS (N/mm)	1.04 ± 0.47	0.97 ± 0.50	0.39 ± 0.06 *3
N.Abnormal Oc/BS (N/mm)	0	1.83 ± 0.44 *4	2.01 ± 0.48 *1
Erosion depth (μm)	18.70 ± 1.66	15.52 ± 1.34 *4	16.53 ± 0.81 *1

Asterisks represent the statistical differences at *p* < 0.05. (*1) between control and LNP + Flo2; (*2) between LNP and LNP + Flo2; (*3) between control and LNP + Flo2 and LNP and LNP + Flo2; and (*4) between control and LNP. N.Ob.Type II/BS: number of type II osteoblasts/bone surface; N.Mu.Oc/BS: number of multinucleated osteoclasts/bone surface; N.Mo.Oc/BS: number of mononucleated osteoclasts/bone surface; Tb.Th: trabecular thickness; O.Th: osteoid thickness.

**Table 4:** Mineralizing parameters of trabecular bone.

Parameter	Control	LNP	LNP + Flo2
BV/TV (%)	7.71 ± 1.36	7.49 ± 1/35	11.68 ± 1/94 *1
ES/BS (%)	39.9 ± 3.9	40.2 ± 7.1	32.3 ± 3.2 *1
MAR (μm/D)	2.58 ± 0.34	2.72 ± 0.28	3.10 ± 0.11 *1
MS/BS (%)	40.9 ± 2.7	38.8 ± 2.4	40.0 ± 2.5
BFR/BS (mm3/mm2/year)	0.38 ± 0.04	0.38 ± 0.04	0.45 ± 0.04 *1
Ob.S/BS (%)	17.8 ± 3.0	19.1 ± 2.0	26.0 ± 4.4 *1
Oc.S/BS (%)	11.2 ± 1.9	9.7 ± 7.5	4.7 ± 2.9 *2

Asterisks represent the statistical differences at *p* < 0.05. (*1) between control and LNP + Flo2 and between LNP and LNP + Flo2; and (*2) between control and LNP + Flo2. BV: bone volume; TV: tissue volume; ES: erosion surface; MAR: mineral apposition rate; MS: mineralizing surface; BFR: bone formation rate; Ob.S: surface area of osteoblasts; Oc.S: surface area of osteoclasts.

## Data Availability

The data presented in this study are available upon request from the corresponding author.

## Abbreviations

The following abbreviations are used in this manuscript.

ALP	alkaline phosphatase
ANOVA	analysis of variance
BMP	bone morphogenetic protein
BS	bone surface
BV	bone volume
Ca	calcium
CL	calcein
CTX	C-terminal telopeptides of type I collagen
dLS	double-labeled surface
ES	eroded surface
Flo2	flotillin-2
Gla	carboxylated osteocalcin
Glu	under-carboxylated osteocalcin
HbA1c	hemoglobin A1c
IP	inorganic phosphorus
LNP	lipid nanoparticle
miRNA	microRNA
MV	microvesicle
Obs	osteoblasts
OCN	osteocalcin
OCs	osteoclasts
SAMP6	senescence-accelerated mice prone 6 strain
SD	standard deviation
Tb.Th	trabecular thickness
TL	tetracycline hydrochloride
TP	total protein
TRACP 5b	tartrate-resistant acid phosphatase-5b
TV	tissue volume
U-Ca	urinary calcium
U-Cre	urinary creatinine
